# Endoscopic vacuum therapy for anastomotic leakage after upper gastrointestinal surgery

**DOI:** 10.1055/a-2102-1691

**Published:** 2023-07-17

**Authors:** Lisanne M. D. Pattynama, Roos E. Pouw, Mark I. van Berge Henegouwen, Freek Daams, Suzanne S. Gisbertz, Jacques J. G. H. M. Bergman, Wietse J. Eshuis

**Affiliations:** 1 Department of Surgery, Amsterdam UMC location University of Amsterdam, Amsterdam, The Netherlands; 2 Department of Gastroenterology and Hepatology, Amsterdam UMC location University of Amsterdam, Amsterdam, The Netherlands; 3 Cancer Treatment and Quality of Life, Cancer Center Amsterdam, Amsterdam, The Netherlands; 4 Amsterdam Gastroenterology Endocrinology Metabolism, Amsterdam, The Netherlands; 5 Department of Gastroenterology and Hepatology, Amsterdam UMC location Vrije Universiteit Amsterdam, Amsterdam, The Netherlands; 6 Department of Surgery, Amsterdam UMC location Vrije Universiteit Amsterdam, Amsterdam, The Netherlands

## Abstract

**Background** Recently, endoscopic vacuum therapy (EVT) was introduced as treatment for anastomotic leakage after upper gastrointestinal (GI) surgery. The aim of this study was to describe the initial experience with EVT for anastomotic leakage after upper GI surgery in a tertiary referral center.

**Methods** Patients treated with EVT for anastomotic leakage after upper GI surgery were included retrospectively (January 2018–June 2021) and prospectively (June 2021–October 2021). The primary end point was the EVT success rate. Secondary end points included mortality and adverse events.

**Results** 38 patients were included (31 men; mean age 66 years): 27 had undergone an esophagectomy with gastric conduit reconstruction and 11 a total gastrectomy with esophagojejunal anastomosis. EVT was successful in 28 patients (74 %, 95 %CI 57 %–87 %). In 10 patients, EVT failed: deceased owing to radiation pneumonitis (n = 1), EVT-associated complications (n = 2), and defect closure not achieved (n = 7). Mean duration of successful EVT was 33 days, with a median of six EVT-related endoscopies. Median hospital stay was 45 days.

**Conclusion** This initial experience with EVT for anastomotic leakage after upper GI surgery demonstrated a success rate of 74 %. EVT is a promising therapy that could prevent further major surgery. More experience with the technique and its indications will likely improve success rates in the future.

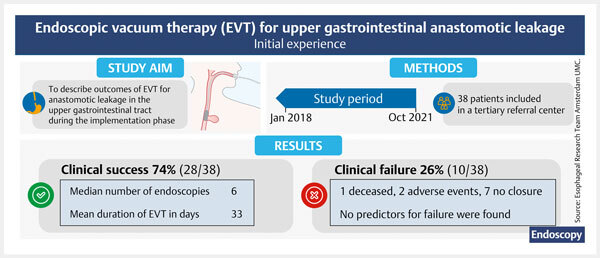

## Introduction

Surgery provides the best chance of cure for esophagogastric cancer 1 2 3 . Anastomotic leakage is one of the most common complications and is associated with severe morbidity and mortality 4 5 .

EVT is a novel endoscopic treatment, based on negative pressure wound therapy, that stimulates wound healing, exudate control, and perfusion 6 . EVT is an effective therapy for anastomotic leakage after colorectal surgery 7 . Recently, EVT was introduced as a treatment modality for anastomotic leakage after upper gastrointestinal (GI) surgery. Results so far, also when compared to stents, are promising with success rates of up to 100 % 8 9 10 ; however, the literature mostly consists of small case series.

In our center, since 2018, EVT has gradually become the treatment of choice for anastomotic leakage after upper GI surgery. The aim of this study was to describe the initial experience with EVT for anastomotic leakage after upper GI surgery in a tertiary referral center.

## Methods

### Patients

All patients treated with EVT for anastomotic leakage after upper GI surgery at Amsterdam UMC between January 2018 and October 2021 were included: retrospectively (January 2018 to June 2021) and prospectively (until October 2021). Data were collected from a prospectively maintained database of patients undergoing upper GI surgery, including salvage resections; details regarding EVT were collected retrospectively. The diagnosis of anastomotic leakage was based on computed tomography (CT) scan findings, such as extraluminal oral contrast, and/or endoscopic findings, such as visualization of a transmural defect.

This study was assessed by the local medical ethics committee, who waived the need for formal ethical review.

### Surgery

Surgery was performed according to the Enhanced Recovery After Surgery protocol and Dutch guidelines for esophagogastric cancer 11 . Surgery was performed minimally invasively, unless an open procedure was warranted. Esophagectomy was preferably performed by a transthoracic approach with two-field lymph node dissection, and gastric conduit reconstruction with an intrathoracic anastomosis (Ivor Lewis). If there was a proximal tumor or radiation field, a cervical anastomosis (McKeown) was performed.

For gastric cancer, total gastrectomies with Roux-en-Y reconstruction were included. When indicated (e. g. large esophagogastric junction tumors), distal esophagectomy and total gastrectomy with Roux-en-Y reconstruction, or esophageal–cardia resection with gastric conduit reconstruction were performed via a left thoracoabdominal approach.

### EVT procedures

EVT procedures were performed with the patients under deep propofol sedation or general anesthesia. The sponge used was the EsoSponge (EsoSPONGE; Braun B. Melsungen, Germany), a polyurethane sponge of 50 mm in length and 13 mm in diameter.

**Fig. 1:**
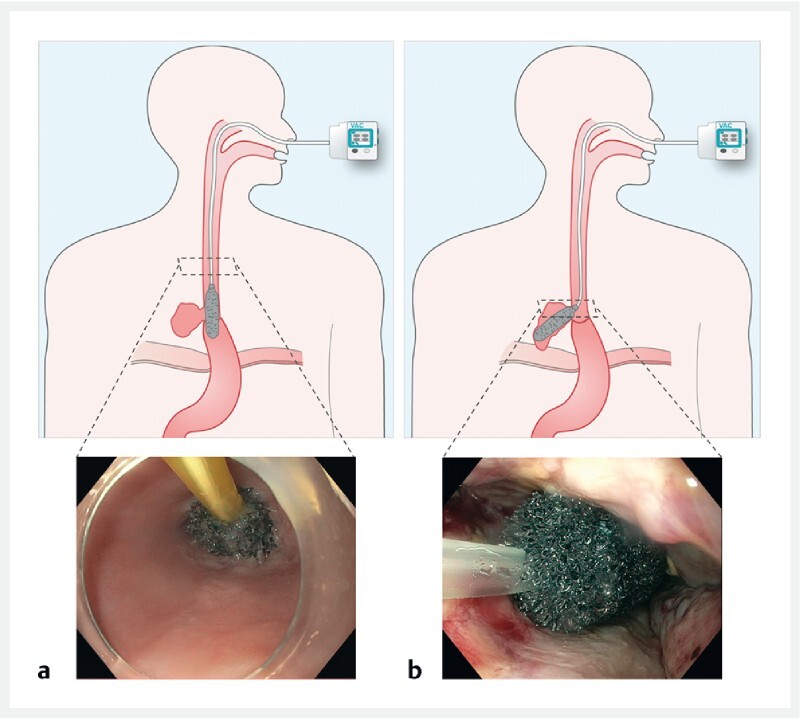
Schematic with accompanying endoscopic view of: **a** the intraluminal technique; **b** the intracavitary technique. Source: Esophageal Research Team Amsterdam UMC.

During the initial endoscopy, the defect and extraluminal cavity were cleaned. The EVT technique was determined by the endoscopist, taking into consideration the defect and cavity width and the extent of the debris. Generally, patients with defects large enough for endoscope passage and big extraluminal cavities initially underwent extraluminal therapy, while patients with small defects and cavities proceeded straight to intraluminal therapy ( Fig. 1 ).

The appropriate sponge size was determined and, if necessary, it was trimmed based on the cavity width. Therapy was considered adequate if the cavity collapsed after application of the vacuum.

Sponge placement was performed using the EsoSponge overtube or a grasping forceps, as preferred by the endoscopist. After sponge placement, the tube of the sponge was guided from the oral cavity to the nose and fixed with a plaster onto the nose. Correct positioning was confirmed under endoscopic vision and vacuum was applied. The pressure of the pump (ActiV.A.C.; 3 M Health Care, St. Paul, Minnesota, USA) was generally −50 mmHg (intracavitary sponge) to −75 mmHg (intraluminal sponge). Intraluminal sponges were exchanged once per week, and intracavitary sponges were exchanged twice per week.

### Outcome parameters

The primary outcome parameter was successful EVT treatment, defined as closure of the defect primarily by EVT. Closure was confirmed by endoscopic inspection and/or CT imaging. Therapy was considered unsuccessful if the defect persisted or increased under EVT or if adequate vacuum therapy was not achieved.

Secondary outcome parameters included additional treatment modalities, adverse events (AEs), and combined in-hospital and 30-day mortality. AEs were defined as any event interfering with the scheduled treatment and were classified by degree of consequences 12 . Incidents were defined as unplanned events not interfering with the planned procedure. For technical complications, a sponge was deemed “dislocated” if an incorrect position was endoscopically observed; minimally visible suction effect on the epithelium was classified as “dysfunction of the vacuum system.” Furthermore, factors possibly associated with the primary outcome were assessed.

### Statistical analysis

Statistical analyses were performed using SPSS Statistics Version 28 (SPSS Inc., Chicago, Illinois, USA). Descriptive data were expressed as numbers with percentages and exact 95 %CI, with median and interquartile rage (IQR) for data with a skewed distribution, and mean (SD) for data with a symmetric distribution. Binomial univariate logistic regression was used to calculate differences between successful and unsuccessful treatment per variable. *P* values were two-sided and considered statistically significant when < 0.05.

## Results

Respectively, 363 and 68 patients underwent esophagectomy and total gastrectomy in the study period, of whom 56 developed anastomotic leakage (13 %, 95 %CI 10 %–17 %) ( **Fig. 1 s** , see online-only Supplementary material). There were 38 patients treated with EVT, who were included in this study. Table 1 details the characteristics of the included patients.

**Table 1:** Baseline characteristics of the 38 patients treated with endoscopic vacuum therapy for anastomotic leakage.

	Total (n = 38)	Esophagectomy (n = 27)	Total gastrectomy (n = 7)	Total gastrectomy with distal esophagectomy (n = 4)
Age, mean (SD), years	66 (9)	65 (10)	68 (10)	71 (7)
Sex, n (%)				
Male	31 (82)	22 (81)	5 (71)	4 (100)
Female	7 (18)	5 (19)	2 (29)	0 (0)
Risk factors for development of anastomotic leakage, n (%)				
Malnutrition 1	28 (74)	20 (74)	5 (71)	3 (75)
Heart failure	4 (11)	2 (7)	1 (14)	1 (25)
Diabetes mellitus (type II)	11 (29)	5 (19)	4 (57)	2 (50)
Hypertension	15 (40)	8 (30)	5 (71)	2 (50)
Renal insufficiency	2 (5)	0 (0)	2 (29)	0 (0)
Steroids	3 (8)	3 (11)	0 (0)	0 (0)
Smoking	29 (76)	20 (74)	5 (71)	4 (100)
Indication for surgery, n (%)				
Adenocarcinoma	30 (79)	20 (74)	6 (86)	4 (100)
Squamous cell carcinoma	5 (13)	5 (19)	0 (0)	0 (0)
MANEC	1 (3)	1 (4)	0 (0)	0 (0)
Adenosquamous carcinoma	1 (3)	1 (4)	0 (0)	0 (0)
Juvenile polyposis	1 (3)	0 (0)	1 (14)	0 (0)
Neoadjuvant therapy, n (%)				
Chemoradiotherapy	26 (68)	26 (96)	0 (0)	1 (25)
Chemotherapy	5 (13)	1 (4)	4 (57)	0 (0)
None	7 (18)	0 (0)	3 (43)	3 (75)
Esophagectomy technique, n (%)				
Ivor Lewis	22 (58)	22 (81)	N/A	N/A
McKeown	5 (13)	5 (19)	N/A	N/A
Surgical approach, n (%)				
Minimally invasive	34 (89)	26 (96)	7 (100)	1 (25)
Open left thoracoabdominal	2 (5)	0 (0)	0 (0)	2 (50)
Open	2 (5)	1 (4)	0 (0)	1 (25)

MANEC, mixed adenoneuroendocrine carcinoma; N/A, not applicable.

1 Malnutrition was defined as underweight (body mass index [BMI] < 18.5 kg/m ^2^ ) and overweight (BMI > 25 kg/m ^2^ ).

### Primary outcome

Successful treatment was achieved in 28 /38 patients (74 %, 95 %CI 57 %–87 %). In 10 patients, EVT failed (26 %, 95 %CI 13 %–43 %), owing to AEs (n = 2), death during (but not due to) EVT (n = 1), and unachieved defect closure (n = 7). The patients with unachieved defect closure underwent additional surgery after a median of 3 (IQR 2–4) EVT-related endoscopies. More details on the patients with unsuccessful EVT are described in **Table 1 s** .

### Secondary outcomes

Prior to EVT, three patients underwent intraluminal stenting (n = 2) and/or resection of the ischemic part of the gastric conduit with re-anastomosis (n = 2). The reason for the use of stenting before EVT was familiarity with the stent and little experience with EVT at that time. The stents prior to EVT were in situ for 5 and 19 days. One patient underwent intraluminal stenting for 5 days in between EVT sessions, because of hesitation regarding vacuum function with a chest tube present. In all of these cases, intraluminal stenting was discontinued because of leakage around the stent.

Two severe AEs occurred because of EVT (5 %, 95 %CI 1 %–18 %): a tracheoesophageal fistula, resulting in a re-operation with repair of the bronchial defect, gastric conduit resection, and cervical esophagostomy; an iatrogenic defect expansion owing to the overtube during a sponge exchange, after which a re-operation with re-anastomosis was performed. Two incidents occurred (5 %, 95 %CI 1 %–18 %): an esophageal ulcer due to the suction catheter and a minor hemorrhage during sponge removal.

The combined mortality rate was 8 % (3 /38; 95 %CI 2 %–21 %). One patient died after 2 weeks of EVT treatment owing to radiation pneumonitis, while improvement of the defect had been observed. One patient died 19 days after completion of successful EVT owing to pulmonary embolism. Another patient died because of acute respiratory distress syndrome 2.5 months after completion of successful EVT. One or more technical complications occurred in 24 patients, including sponge rupture during removal (n = 4), dysfunction of the vacuum system (n = 6), and sponge dislocation (n = 16), of which two were dislodged by the patient pulling the suction catheter.

Table 2 displays a comparison of the clinical factors for successful and unsuccessful treatment. In univariate analysis, no factor showed a significant association with the outcome. Among the successful EVT treatments, a median of six (IQR 3–11) EVT-related endoscopies were performed during a mean (SD) of 33 (24) days of treatment ( **Table 2 s** ).

**Table 2:** Comparison of the clinical characteristics of the patients in the successful and unsuccessful endoscopic vacuum therapy (EVT) groups.

	All patients (n = 38)	Successful treatment (n = 28)	Unsuccessful treatment (n = 10)	*P* value
Anastomosis level and type, n (%)				
Cervical esophagogastric	5 (13)	3 (11)	2 (20)	0.46
Intrathoracic esophagogastric	22 (58)	17 (61)	5 (50)	0.43
Intrathoracic esophagojejunal	5 (13)	4 (14)	1 (10)	0.73
Intra-abdominal esophagojejunal	6 (16)	4 (14)	2 (20)	0.67
Time to diagnosis, mean (SD), days	9 (4)	9 (4)	7 (4)	0.07
Time from diagnosis to treatment, median (IQR), days	0 (0–1)	0 (0–1)	0 (0–1)	0.78
CRP at diagnosis, median (IQR), mg/L	239 (158–330)	228 (149–330)	300 (208–417)	0.10
WBC at diagnosis, mean (SD), × 10 ^6^ /L	14 (5)	15 (5)	12 (4)	0.13
Defect dehiscence of circumference *,* n (%)				
Small ( < 10 %)	17 (45)	11 (39)	6 (60)	0.26
Intermediate (10 %–40 %)	12 (32)	12 (43)	0 (0)	> 0.99
Large ( > 40 %)	9 (24)	5 (18)	4 (40)	0.17
EVT technique at first endoscopy, n (%)				0.78
Intraluminal	35 (92)	26 (93)	9 (90)	
Intracavitary	3 (8)	2 (7)	1 (10)	
Presence of collection(s) and additional percutaneous or surgical drainage, n (%)	16 (42)	12 (43)	4 (40)	0.70

IQR, interquartile range; CRP, C-reactive protein; WBC, white blood cell count.

## Discussion

In this study, the initial experience with EVT for anastomotic leakage after upper GI surgery in a tertiary referral center is described. EVT appeared efficient and safe in this implementation phase, with a success rate of 74 % and an AE rate of 5 %.

Although these results correspond with previous studies, with success rates of 60 % to 100 % and AE rates of 0 % to 6 %, some studies found higher success rates 8 9 10 13 14 . These studies included tailor-made EVT techniques, whereas the cohort described in this paper used only the EsoSponge; however, we believe that the relatively low success rate is due mainly to the implementation phase. During this implementation phase, we felt a learning curve was present, as reported previously by Reimer et al. 15 . The lessons learnt in this phase included the prevention of possible causes of failure, such as dislocation of the sponge.

In this cohort, a relatively high number of sponge ruptures occurred. To prevent this, we have developed a technique to safely remove the sponge without rupture. Before sponge removal, a distal attachment cap is placed on the endoscope and, subsequently, the endoscope is maneuvered between the sponge and the mucosa to carefully separate the sponge from the (esophageal) wall. After all sides of the sponge have been loosened, the sponge is easily removed, without needing to firmly pull it, and rupture is prevented.

Comparison of the EVT success and failure groups might indicate factors that contribute to therapy failure, which could help determine the best indications for EVT. In this cohort, no significant differences were found between these groups; however, because of the small sample sizes in the subgroups, it is possible that factors that actually do contribute to therapy failure were not statistically significant. For example, more esophagojejunal anastomoses, shorter time to diagnosis, higher C-reactive protein (CRP) at diagnosis, and more “intermediate” and “large” defects are striking in the failure group and could possibly be predictors for therapy failure. Book et al. 16 showed similar results. They identified 116 patients treated with EVT and found use of the intracavitary technique at first endoscopy, more complications, and higher CRP and WBC count at diagnosis significantly influenced EVT failure. Jung et al. 17 found a significant influence of neoadjuvant treatment, intraluminal technique, and increased CRP at diagnosis on therapy failure in 119 patients.

This study describes the outcomes of consecutive patients treated during the implementation phase of EVT into the routine treatment of anastomotic leakage at a tertiary referral center. The protocol was optimized during this implementation phase and some important lessons were learned. For example, the use of a grasping forceps to introduce the sponge under endoscopic visualization replaced the overtube as the preferred placement technique. In addition, intracavitary EVT was found to be of great importance for adequate treatment when a cavity was present. The intraluminal technique should be applied only when a cavity is small enough to collapse with the vacuum.

Close and efficient collaboration between the surgical and gastroenterological departments is also essential to optimize individualized management of patients with EVT. The implementation of EVT can be associated with logistic challenges. In 45 % (95 %CI 29 %–61 %) of the patients in our cohort, the first sponge placement occurred outside of the regular endoscopy program, requiring flexibility of both the endoscopy and anesthesiology teams. Moreover, the regular sponge exchanges with the patient under deep propofol sedation or general anesthesia are difficult to schedule. Therefore, having a dedicated team of trained interventional endoscopists and designated endoscopy slots is helpful in organizing optimal care for EVT patients.

This study has several strengths. Because this study describes a consecutive cohort of patients in the implementation phase of EVT, it allows us to share important lessons on EVT treatment. Furthermore, all patients were treated by a group of experienced interventional endoscopists, in close collaboration with the surgical team. Lastly, to our knowledge, this study is the first to compare the characteristics of the EVT failure and success groups in patients with anastomotic leakage only.

This study also has limitations. First, as there is no control group, no conclusions can be drawn regarding the comparison of EVT with other treatment options for anastomotic leakage. Second, owing to the mostly retrospective nature of the study, selection and information bias could have occurred.

Currently, our group is working on a white book with technical and logistic tips and tricks to facilitate EVT implementation in other centers, where implementation may be hampered by logistical challenges and difficulties in obtaining adequate training. Wider application of the technique, as well as prospective registration, will enable future studies in larger cohorts of patients. Moreover, this will enable the evaluation of new devices for EVT, such as open-pore film drainage, which provides the benefits of a longer small-lumen device and less sponge ingrowth 18 , and the VACStent, which combines the sealing effect of a stent with negative pressure wound therapy at the defect site, keeping the stent in place with the vacuum and allowing for oral intake 19 . Furthermore, the pre-emptive use of EVT may help prevent anastomotic leakage after upper GI surgery 20 .

In conclusion, EVT is a paradigm shifting treatment for anastomotic leakage after upper GI surgery, with a success rate of 74 % in this study during implementation of the technique. Currently many aspects of EVT are experience-based, rather than evidence-based. Future studies will provide more evidence on the best indications and techniques for EVT, which, along with more experience, will likely improve outcomes in the future.

## Fig. 1 s, Tables 1 s–2 s,

Supplementary material Zusatzmaterial
