
JOURNAL INFORMATION
==============================
NLM Title Abbreviation: Endoscopy
Journal ID: Endoscopy
NLM Journal ID: 0215166

Endoscopy

ISSN: 0013-726X
EISSN: 1438-8812

ARTICLE INFORMATION
==============================
PMCID: PMC12419955
PMID: 38917968
DOI: 10.1055/a-2350-8252
Article version: 1
Subjects: Correction

Correction: Endoscopic vacuum therapy for anastomotic leakage after upper gastrointestinal surgery

http://orcid.org/0000-0003-4136-4778 Pattynama Lisanne M. D. 1 2 3 4
Pouw Roos E. 3 5
van Berge Henegouwen Mark I. 1 3
Daams Freek 3 6
Gisbertz Suzanne S. 1 3
Bergman Jacques J. G. H. M. 2
Eshuis Wietse J. 1 3
1 Department of Surgery, Amsterdam UMC location University of Amsterdam, Amsterdam, The Netherlands
2 Department of Gastroenterology and Hepatology, Amsterdam UMC location University of Amsterdam, Amsterdam, The Netherlands
3 Cancer Treatment and Quality of Life, Cancer Center Amsterdam, Amsterdam, The Netherlands
4 Amsterdam Gastroenterology Endocrinology Metabolism, Amsterdam, The Netherlands
5 Department of Gastroenterology and Hepatology, Amsterdam UMC location Vrije Universiteit Amsterdam, Amsterdam, The Netherlands
6 Department of Surgery, Amsterdam UMC location Vrije Universiteit Amsterdam, Amsterdam, The Netherlands
Corresponding author Wietse J. Eshuis, MD, PhD Department of SurgeryAmsterdam UMC location University of AmsterdamMeibergdreef 9AmsterdamThe Netherlandsw.j.eshuis@amsterdamumc.nl
Electronic publication date: 2024 Jun 25
Publication date: 2023 Nov
Volume: 55
Issue: 11
First page: C11
Last page: C11

Copyright: The Author(s). This is an open access article published by Thieme under the terms of the Creative Commons Attribution License, permitting unrestricted use, distribution, and reproduction so long as the original work is properly cited. (https://creativecommons.org/licenses/by/4.0/)
Copyright year: 2023
Copyright holder: The Author(s).
License: This is an open-access article distributed under the terms of the Creative Commons Attribution License, which permits unrestricted use, distribution, and reproduction in any medium, provided the original work is properly cited.
License URL: https://creativecommons.org/licenses/by/4.0/

Erratum for: Endoscopic vacuum therapy for anastomotic leakage after upper gastrointestinal surgery 55 11 17 7 2023 1019 1025 Endoscopy 10.1055/a-2102-1691 PMC10602657 37253387

==============================
Correction

Correction: Endoscopic vacuum therapy for anastomotic leakage after upper gastrointestinal surgery Pattynama LMD , Pouw RE, van Berge Henegouwen MI et al. Endoscopic vacuum therapy for anastomotic leakage after upper gastrointestinal surgery. Endoscopy 2023; 55: 1019–1025 . In the above-mentioned article, the name of the author Mark I. van Berge Henegouwen has been corrected. This was corrected in the online version on June 24, 2024.

