# Supplementary material for: Endoscopic vacuum therapy for anastomotic leakage after upper gastrointestinal surgery
Source: Endoscopy. 2023 Jul 17;55(11):1019–25. doi: 10.1055/a-2102-1691 (PMC10602657; doi:10.1055/a-2102-1691)
Supplement: Supplementary file 1 — Supplementary material [file 22460supmat_10-1055-a-2102-1691.pdf]

Supplementary material

Endoscopic vacuum therapy for anastomotic leakage after upper gastrointestinal surgery

L.M.D. Pattynama, R.E. Pouw, M.I. van Berge Henegouwen, F. Daams, S.S. Gisbertz, J.J.G.H.M. Bergman, W.J. Eshuis

Figure 1s. Study flowchart between January 2018 and October 2021.

Abbreviations: AL, anastomotic leakage; EVT, endoscopic vacuum therapy.

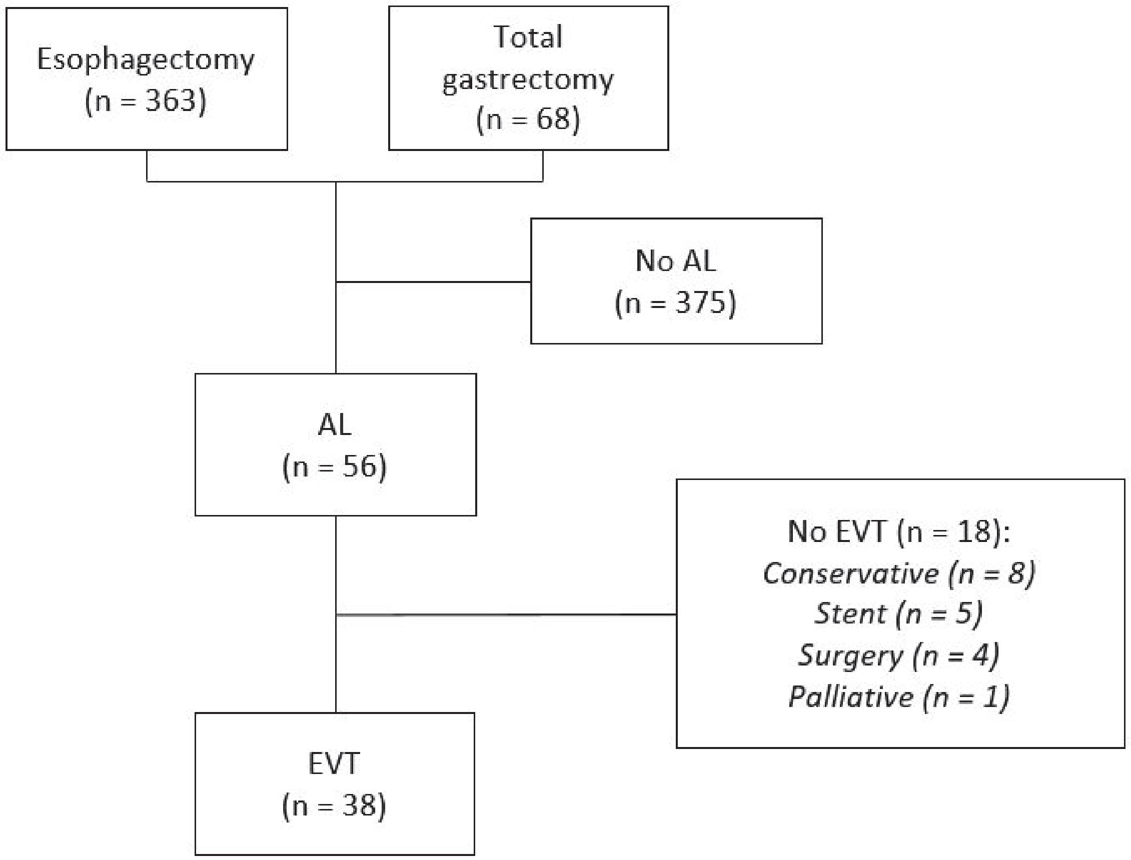

Supplementary material

Table 1s. Details on unsuccessful treatments

	Reason of EVT failure	EVT-related endoscopies, *n*	Initial surgery	Anastomosis	Additional surgery	Preservation of continuity
1	Deceased due to radiation pneumonitis	2	McKeown esophagectomy	Cervical esophago-gastric	N/A	N/A
2	Tracheo-esophageal fistula	5	Ivor Lewis esophagectomy	Thoracic esophago-gastric	Disconnection of anastomosis and construction of cervical esophagostomy	No
3	Iatrogenic defect expansion due to overtube	1	Ivor Lewis esophagectomy	Thoracic esophago-gastric	Re-anastomosis	Yes
4	Enlargement of defect during EVT	4	Ivor Lewis esophagectomy	Thoracic esophago-gastric	Re-anastomosis	Yes
5	Enlargement of defect during EVT in combination with recurrent sponge dislocation	3	McKeown esophagectomy	Cervical esophago-gastric	Disconnection of anastomosis and construction of cervical esophagostomy	No
6	Early leakage with rapid deterioration under EVT in combination with proximally dislocated sponge	2	Total gastrectomy with Roux-en-Y reconstruction	Esophago-jejunostomy	Disconnection of anastomosis and construction of cervical esophagostomy	No
7	Early leakage with rapid deterioration under EVT in combination with proximally dislocated sponge	3	Total gastrectomy with Roux-en-Y reconstruction	Esophago-jejunostomy	Disconnection of anastomosis and construction of cervical esophagostomy	No
8	Enlargement of defect during EVT	5	Open total gastrectomy with Roux-en-Y reconstruction	Esophago-jejunostomy	Disconnection of anastomosis and construction of cervical esophagostomy	No
9	Partly ischemic gastric conduit	2	Ivor Lewis esophagectomy	Thoracic esophago-gastric	Disconnection of anastomosis and construction of cervical esophagostomy	No
10	Partly ischemic gastric conduit	2	Open Ivor Lewis esophagectomy	Thoracic esophago-gastric	Disconnection of anastomosis and construction of cervical esophagostomy	No

Supplementary material

Table 2s. Treatment characteristics in all patients, and in the successful and unsuccessful EVT groups

	**Total (n = 38)**	**Successful treatment (n = 28)**	**Unsuccessful treatment (n = 10)**
Number of EVT-related endoscopies, *median (IQR)*	4 (3-8)	6 (3-11)	3 (2-4)
Duration of EVT, *mean (SD)*	27 (23)	33 (24)	10 (8)
Length of hospital stay in days‡, *median (IQR)*	45 (31-63)	40 (28-65)	53 (35-62)
ICU-stay, *n (%)*	14 (37)	17 (61)	7 (70)
Length of ICU-stay in days, *median (IQR)*	13 (4-40)	13 (4-50)	12 (5-37)
Stenosis requiring dilation during 6 months follow-up, *n (%)*	7/36 (19)	6/27 (22)	1/9 (11)

Abbreviations: EVT, endoscopic vacuum therapy; SD, standard deviation; IQR, interquartile range; ICU, intensive care unit.

‡Time from surgery to discharge or death.

 | © 2023. The author(s).
